# Genome-wide characteristics and potential functions of circular RNAs from the embryo muscle development in Chengkou mountain chicken

**DOI:** 10.3389/fvets.2024.1375042

**Published:** 2024-05-30

**Authors:** Yang Zhang, Haiwei Wang, Xingqi Li, Chaowu Yang, Chunlin Yu, Zhifu Cui, Anfang Liu, Qigui Wang, Lingbin Liu

**Affiliations:** ^1^College of Animal Science and Technology, Southwest University, Chongqing, China; ^2^Chongqing Academy of Animal Sciences, Chongqing, China; ^3^Animal Breeding and Genetics Key Laboratory of Sichuan Province, Sichuan Animal Science Academy, Chengdu, China

**Keywords:** circRNA, muscle development, Chengkou mountain chicken, RNA sequencing, transcriptome

## Abstract

The Chengkou mountain chicken, a native Chinese poultry breed, holds significant importance in the country’s poultry sector due to its delectable meat and robust stress tolerance. Muscle growth and development are pivotal characteristics in poultry breeding, with muscle fiber development during the embryonic period crucial for determining inherent muscle growth potential. Extensive evidence indicates that non-coding RNAs (ncRNAs) play a regulatory role in muscle growth and development. Among ncRNAs, circular RNAs (circRNAs), characterized by a closed-loop structure, have been shown to modulate biological processes through the regulation of microRNAs (miRNAs). This study seeks to identify and characterize the spatiotemporal-specific expression of circRNAs during embryonic muscle development in Chengkou mountain chicken, and to construct the potential regulatory network of circRNAs-miRNA-mRNAs. The muscle fibers of HE-stained sections became more distinct, and their boundaries were more defined over time. Subsequent RNA sequencing of 12 samples from four periods generated 9,904 novel circRNAs, including 917 differentially expressed circRNAs. The weighted gene co-expression network analysis (WGCNA)-identified circRNA source genes significantly enriched pathways related to cell fraction, cell growth, and muscle fiber growth regulation. Furthermore, a competitive endogenous RNA (ceRNA) network constructed using combined data of present and previous differentially expressed circRNAs, miRNA, and mRNA revealed that several circRNA transcripts regulate *MYH1D*, *MYH1B*, *CAPZA1*, and *PERM1* proteins. These findings provide insight into the potential pathways and mechanisms through which circRNAs regulate embryonic muscle development in poultry, a theoretical support for trait improvement in domestic chickens.

## Introduction

Chicken meat is a prominent consumer livestock product globally, with its consumption steadily rising (1). In China, an increasing number of consumers favor local meat breeds. However, local breeds such as the Chengkou mountain chicken are encountering challenges in meeting the demand for high-quality poultry due to their slow growth and development. Consequently, the focus of China’s poultry industry has always been on improving chicken production and ensuring high-quality meat (2).

Skeletal muscle, as the predominant constituent of animal meat products, significantly influences poultry meat yield by virtue of its growth and development (3). The activation of skeletal myogenesis is governed by the myokines *Myf5*, which are present in cells located in the dorsomedial portion of the somites (4). Upon interaction with neural crest cells carrying *Wnt1*, *Myf5* triggers expression in the dermis, leading to the downregulation of *Pax3* expression and the subsequent formation of the primary myotome (5). The process of muscle fiber growth and development is intricate and can be categorized into three stages: early embryonic, late embryonic, and postnatal (6). During the early embryonic stage, monocytic muscle cells, originating from *Pax3^+^* dermal cell progenitors, align along the entire cranio-caudal length of the somite to form the initial sarcomere (7). Subsequently, in the late embryonic stage, embryonic myoblasts fuse into myotubes, and myogenic progenitors differentiate into multinucleated myofibers under the regulation of *MRF4* (8). As the developmental process unfolds, muscle cells produce primary and secondary muscle fibers, with satellite cells gradually forming to supply nuclei for the growing muscle fibers (9). After birth, the muscle fibers mature, and satellite cells enter a quiescent state, reactivating only to repair damaged muscle fibers (10). Enhanced insight into the intricate developmental regulatory network of embryonic skeletal muscle can offer more precise guidance in poultry breeding, thereby improving the economic benefits of breeding.

Circular RNAs (circRNAs) discovered in 1976 are single-stranded circular transcripts that are important in muscle development (11, 12). Trans-splicing without a 5′ cap and 3′poly (A) tail produces circRNAs, which have a longer half-life than linear mRNA (13). Ouyang et al. (14) showed that circRNAs are abundant and dynamically expressed during chick embryonic muscle development. CircRNAs affect cell proliferation in skeletal muscle development (15). An *in vivo* experiment showed that circZfp609, a gene marker of *ZNF609*, inhibits myogenic differentiation by sponging miR-194-5p (16). The isolated circZfp609 inhibits *BCLAF1* and affects the expression of monoclonal antibody to *Myf5* and *MyoG*. Chen et al. (17) showed that overexpressing circCLTH promotes muscle cell differentiation and fusion in buffalo. Furthermore, Liu et al. (18) showed that circARID1A regulates skeletal muscle regeneration in mice by acting as a sponge for miR-6368. Undoubtedly, circRNAs play a pivotal role in muscle development, and investigating their regulatory mechanism is imperative for enhancing poultry meat production performance.

The identification and regulatory functions of circRNAs in Chengkou mountain chicken remain poorly understood. In a previous study, we examined the mechanism of embryonic muscle development in Chengkou mountain chicken using transcriptomes (19). In this study, we aim to elucidate the specific functions of circRNAs in muscle development and explore potential regulatory pathways by analyzing differentially expressed circRNAs at various stages of embryonic muscle development and predicting associated regulatory pathways.

## Methods

### Experimental animals and materials

In this study, Chengkou mountain chicken embryos were used as experimental animals and were purchased from Chongqing Xuanpeng Agricultural Development Co., Ltd. A total of 200 eggs were incubated following the conventional incubation procedure (37.8°C, 55% humidity) and embryos were harvested on Day 12, 16, 19, and 21 of incubation. In order to maintain uniformity across samples from the four periods, we opted for leg muscles as the primary sample due to their ease of collection during the embryonic period. We ensured comprehensive collection of the leg muscles, obtaining three biological replicates per period and stored them at −80°C. Twelve embryonic skeletal muscle samples were fixed with 4% paraformaldehyde and stored at 4°C for histological observation.

### Phenotypic identification of different stages of muscle development

After paraffin embedding, the tissue samples were stained with hematoxylin and eosin, and the muscle fibers were imaged using an OLYMPUS microscope imaging system (Olympus, Tokyo, Japan). For each picture, 30 muscle fibers were randomly selected (19).

### cDNA library construction and circRNA sequencing

Total RNA was extracted from the muscles of the four periods according to the instruction method of Trizol reagent (TaKaRa, Dalian, China), and the concentration and purity of RNA were determined using a NanoDrop microspectrophotometer (Thermo Fisher Scientific, MA, United States). After removal of ribosomal RNA (rRNA) (Epicentre, United States), linear RNA was digested with RNase R enzyme (QIAGEN, Germany) to obtain circular RNA. The fragmented RNA was then treated with a fragmentation buffer to generate short fragments. Random hexamers were used to synthesize the first strand of cDNA, and a buffer, dNTPs, RNase H, and DNA polymerase I (QIAGEN, Germany) were added to synthesize the second strand of circular RNA using the linear RNA as a template. Purification was performed using a QiaQuick PCR kit (QIAGEN, Germany), followed by elution with EB buffer, end repair, sequential addition of base A and sequencing adapter, and recovery of the target fragment by agarose gel electrophoresis. The target fragments were then amplified by PCR to complete the library preparation. The samples were subsequently sent to Gene Denovo Biotechnology Co., Ltd. (Guangzhou, China) for sequencing using Illumina HiSeqTM 2,500 (Illumina, CA, United States).

### Identification and statistics of circRNAs

To ensure data quality, raw reads containing adapter sequences with more than 10% N were removed. The remaining reads were further filtered to remove low-quality reads, where the number of bases with a quality value of *Q* ≤ 10 accounted for more than 50% of the entire read. The resulting high-quality (HQ) clean reads were then matched to the ribosome database using Bowtie2 in the Hisat2 tool (20, 21). The ribosome-mapped reads were removed, leaving only the unmapped reads for subsequent analysis. These unmapped reads were then aligned to the reference genome. The Find_circ software was used to identify circRNAs, and the resulting circRNA identification results were filtered to obtain highly credible circRNAs (22). A difference in circRNA with a | log2 (FC) | > 1 and FDR (false discovery rate) < 0.05 is defined as significant.

### Function enrichment analysis of differentially expressed circRNAs

Using DAVID25 software, we mapped all of the source genes to the Gene Ontology database.^1^ We then calculated the number of genes associated with each GO entry through hypergeometric inspection, defining the background of source genes and determining significant enrichment of GO terms relative to the genome. KEGG pathway enrichment of source genes was performed using KOBAS v2.0.

### Time series analysis

Utilizing the Short Time-Series Expression Miner (STEM) software,^2^ time-series clustering analysis of differentially expressed circRNAs was performed to elucidate various gene expression patterns during the embryonic stage. The polygenic screening required a minimum of 2 variants and allowed for a maximum of 20 trends. Data normalization was carried out using log2 (RPM), with a *p* < 0.05 considered indicative of a reliable trend.

### WGCNA analysis

The WGCNA R package was used to cluster genes with similar expression patterns, investigate the association between modules and specific phenotypes or traits, and examine the expression patterns of multiple genes (23). The soft threshold was set to 2, with all other settings remaining at their default values. A *p*-value of <0.05 was considered indicative of module association.

### The ceRNA network construction

Utilizing the Cytoscape software,^3^ we drew ceRNA networks and predicted potential relationships among circRNAs, mRNAs, and miRNAs using the miRDB^4^ online database. The construction of the ceRNA network followed the Cytoscape network construction template, incorporating mRNA and miRNA data from our team’s previous research results (19, 24–26). The screening criteria in miRDB included only functional miRNAs, while excluding targets with a prediction score of less than 80, and miRNAs with more than 2,000 predicted targets in the genome.

### Quantitative verification

Here, we selected six circRNAs to verify the sequencing results via RT-qPCR. Primers were designed using Primer Premier, as shown in Supplementary Table S3. The specific PCR method was based on previous experiments conducted by our team (19).

### Statistical analysis

The data were processed using SPSS 20.0 (SPSS Inc., United States) software and presented as mean ± standard deviation (mean ± SD). A comparison between the two groups was conducted using the unpaired t-test and Duncan multiple range test. Graphs were generated using GraphPad Prism 9 (San Diego, CA, United States). *p* < 0.05 was considered statistically significant.

## Results

### Phenotypes at different stages of muscle development

**Figure 1:**
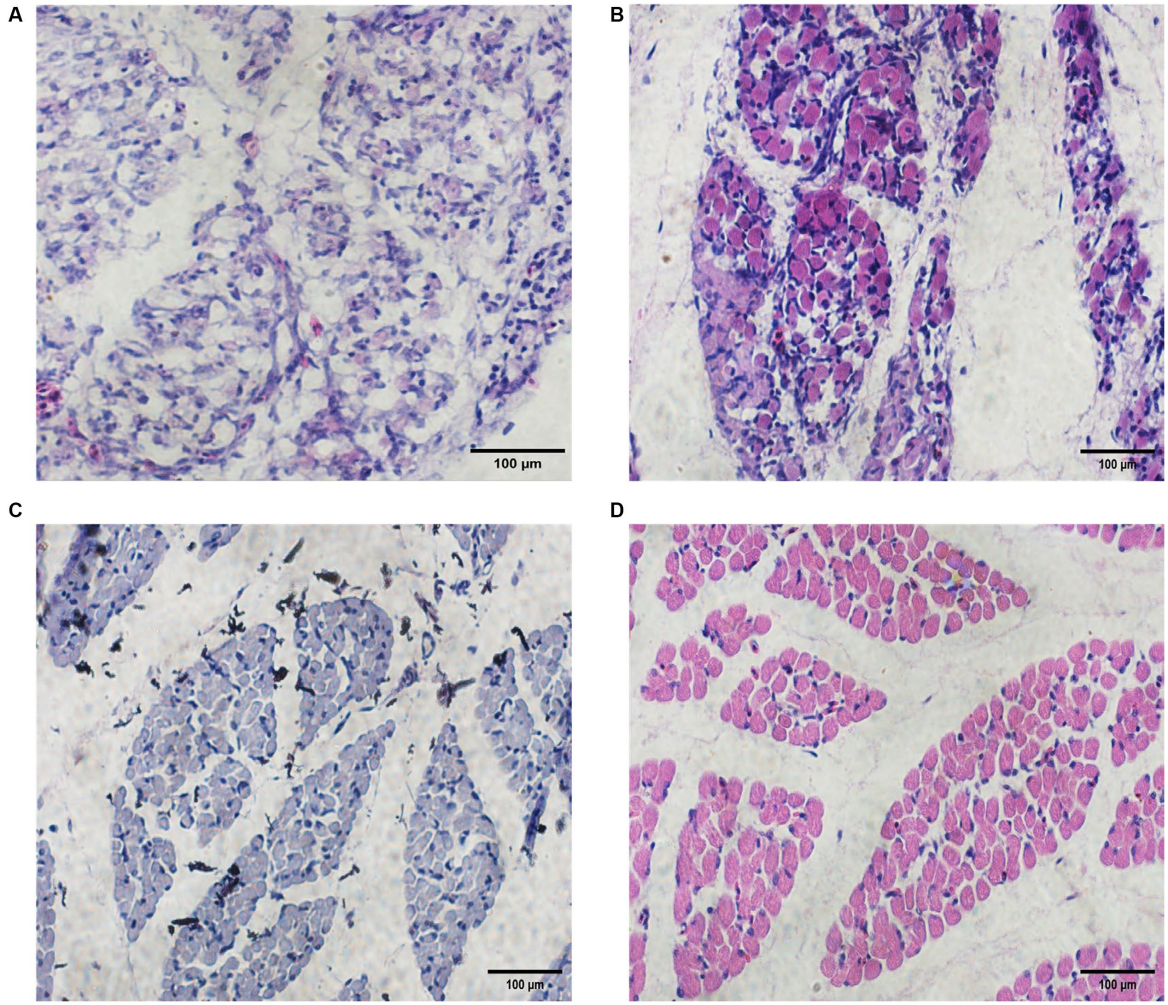
Embryonic muscle sections and muscle fiber diameters of Chengkou mountain chicken. **(A–D)** Histological characteristics in E12, E16, E19, and E21. Scale bar = 100 μm.

We stained sections of Chengkou mountain chicken embryonic muscles at four stages and compared the muscle fibers to determine muscle development at the respective stages (Figure 1). Hematoxylin-Eosin sections showed that on Day 12 (E12), the muscle fibers were compounded. By Day 16 (E16), the muscle fibers’ outline had emerged and their diameter had notably expanded. At Day 19 (E19), the differentiation of myofibers became more pronounced, and their diameter continued to increase. The basic structure of the muscle fibers was visible, becoming clearest by Day 21 (E21) when the fiber structure had fully matured and could be clearly identified. The analysis revealed a gradual increase in muscle fiber diameter over the course of time (Supplementary Figure S1). The HE results showed the basic rules of muscle growth in Chengkou mountain chickens, providing a preliminary basis for subsequent analyses.

### Overview and quality assessment of sequencing data

The 12 sequenced cDNA libraries (E12-1, E12-2, E12-3, E16-1, E16-2, E16-3, E19-1, E19-2, E19-3, E21-1, E21-2 and E21-3) generated 1,334,509,224 high-quality clean reads after filtering out reads with adapter sequences, >10% N ratio, and low-quality bases (Supplementary Table S1). After filtering, the base quality of all samples (Q20 values) was greater than 98% and the GC content was approximately 45%, with a balanced base composition clean reads (Supplementary Figure S2). Approximately 93% of the reads were mapped to the chicken reference genome (Table 1). The suspected circRNA reads (anchor reads) were subsequently analyzed by comparing them with the reference genome using bowtie to identify circRNAs (Table 2).

**Table 1:** Statistical table of the alignment of total reads to the reference genome.

Sample	Total reads	Mapped reads	Mapping ratio
E12-1	110,098,734	105,324,562	95.66%
E12-2	81,283,742	77,598,849	95.47%
E12-3	72,787,674	69,552,509	95.56%
E16-1	80,878,246	77,522,368	95.85%
E16-2	146,786,694	140,216,208	95.52%
E16-3	128,160,934	122,074,700	95.25%
E19-1	113,707,428	108,004,836	94.98%
E19-2	129,016,962	122,751,154	95.14%
E19-3	115,085,998	109,631,848	95.26%
E21-1	89,152,220	83,829,192	94.03%
E21-2	139,382,784	130,704,097	93.77%
E21-3	108,925,286	102,379,331	93.99%

**Table 2:** Table of comparison between anchor reads and the chicken reference genome.

Sample	Anchors numbers	Mapped anchors	Mapping ratio
E12-1	9,548,344	6,280,396	65.77%
E12-2	7,369,786	4,942,628	67.07%
E12-3	6,470,330	4,438,724	68.60%
E16-1	6,711,756	4,416,778	65.81%
E16-2	13,140,972	8,335,920	63.43%
E16-3	12,172,468	7,726,738	63.48%
E19-1	11,405,184	6,458,868	56.63%
E19-2	12,531,616	7,135,557	56.94%
E19-3	10,908,300	6,706,731	61.48%
E21-1	10,646,056	5,461,306	51.30%
E21-2	17,357,374	8,385,928	48.31%
E21-3	13,091,910	6,774,769	51.75%

### Investigating the molecular features of circRNAs in embryonic muscle of the Chengkou mountain chicken

**Figure 2:**
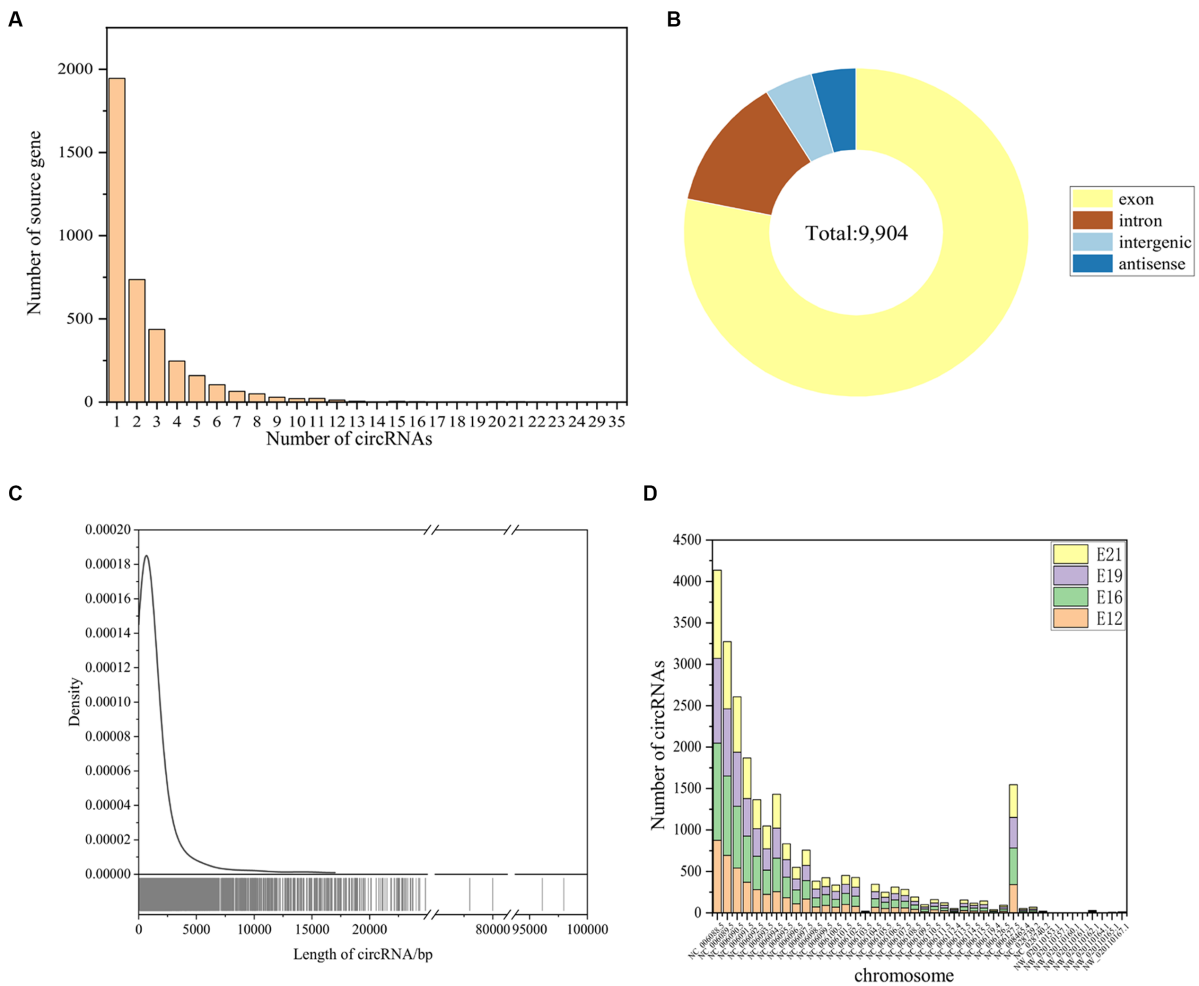
Identification of circRNAs. **(A)** Source genes of the identified circRNAs. **(B)** Type identification of all novel circRNAs. **(C)** Length distribution of all novel circRNAs. **(D)** The distribution of identified circRNAs in different chromosomes.

A total of 9,904 novel circRNAs were identified from 3,273 genes, with 1,945 source genes producing only one circRNA and the most, 35, coming from the same gene (Figure 2A). CircRNAs were predominantly derived from exons, accounting for 78.27%, followed by introns (13.05%), intergenic regions (4.48%), and antisense circRNAs (4.20%) (Figure 2B). The identified circRNAs had a range of lengths, from 73 bp to 97,968 bp (Figure 2C). Most of these circRNAs were derived from chicken chromosome one (NC_006088.5) (Figure 2D).

### Differential expression analysis of circRNAs

**Figure 3:**
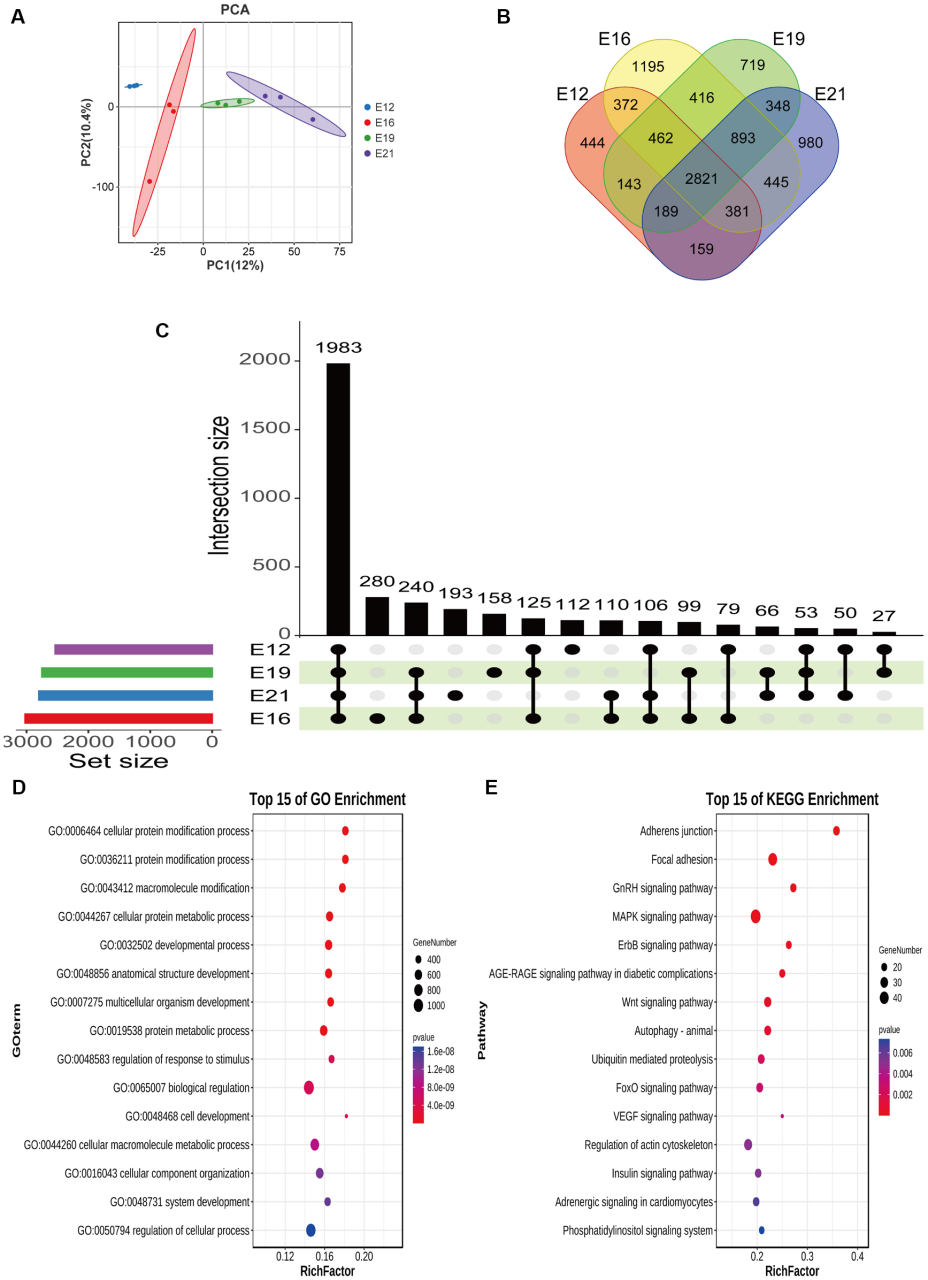
Differential expression analysis of circRNAs. **(A)** Principal component analysis of samples. **(B)** Distribution of circRNA number in different periods. **(C)** Upset plot of circRNA-derived genes in different periods. **(D)** GO analysis of circRNA-derived genes at the same intersection in four periods. **(E)** KEGG analysis of circRNA-derived genes at the same intersection in four periods.

**Figure 4:**
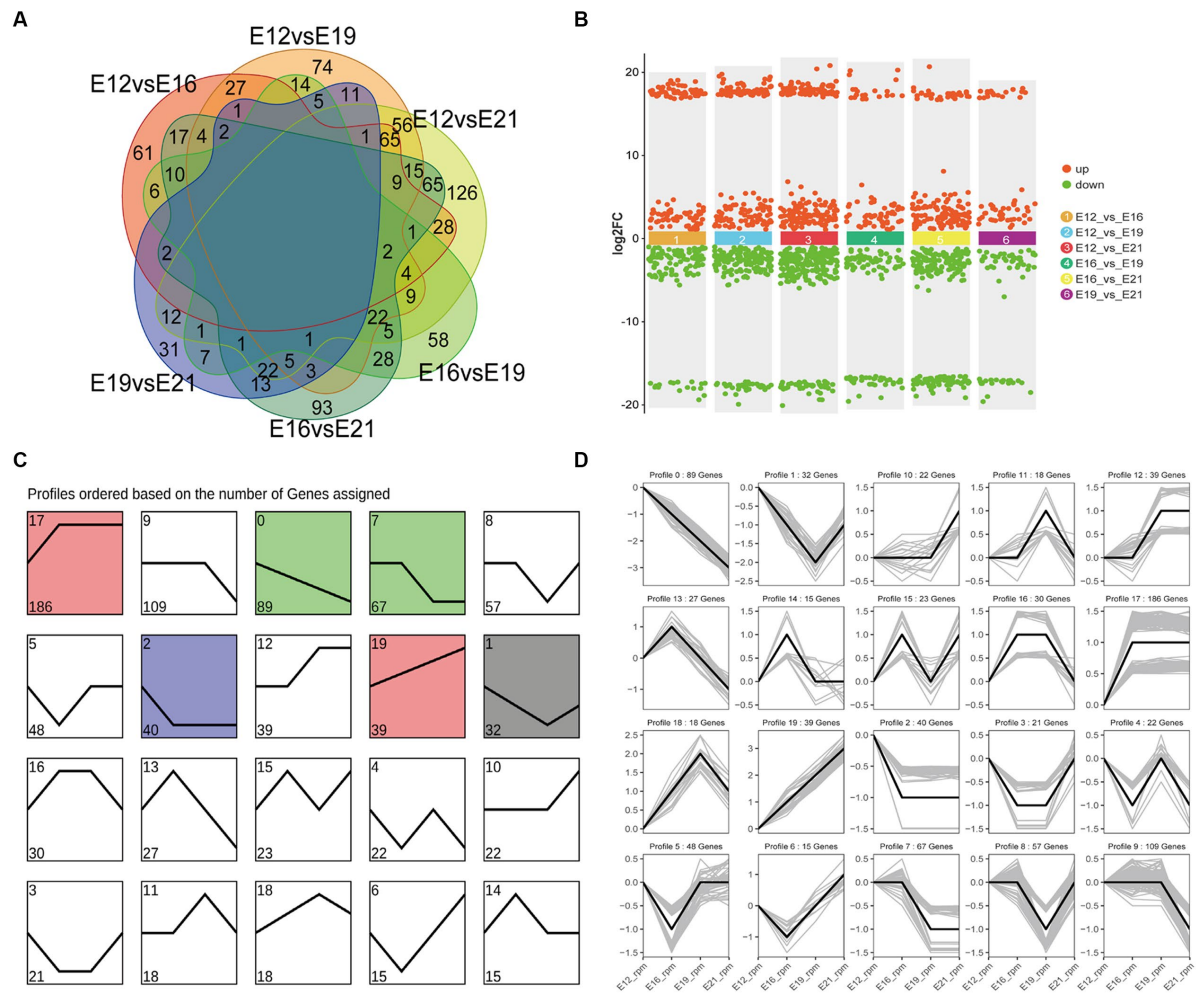
Analysis of differential circRNAs in four periods. **(A)** The number of differentially expressed circRNAs was compared between the four periods. **(B)** Up-and down-regulated circRNA analysis. **(C)** Distribution trend of differential circRNA expression, color means significant difference (*p* < 0.05), gray means not significant (*p* > 0.05). **(D)** Time line of differential circRNAs.

A principal component analysis (PCA) showed that the 12 samples clustered according to the biological repeats of different time nodes (Figure 3A), indicating the biological repeatability and significance of between-group differences. There were 444 circRNAs expressed only at E12, 1,195 only at E16, 719 only at E19, and 980 only at E21 (Figure 3B). A total of 1,983 common circRNA-derived genes were identified across the four periods (Figure 3C), which were mainly associated with protein binding, intracellular parts, and cellular protein modification processes (Figure 3D). Pathway analysis revealed that these genes were mainly implicated in focal adhesion, the *GnRH* signaling pathway, and the *MAPK* signaling pathway (Figure 3E). A total of 917 novel circRNAs exhibiting differential expression (|log2(FC)| > 1, FDR < 0.05) were identified through pairwise comparison of the four periods (Figure 4A), including 240 in E12 vs. E16, 330 in E12 vs. E19, 450 in E12 vs. E21, 174 in E16 vs. E19, 320 in E16 vs. E21 and 118 in E19 vs. E21 (Supplementary Table S1). In comparison to E12, E16, and E19, E21 had 121, 150, and 196 up-regulated circRNAs, respectively, and 119, 180, and 254 down-regulated circRNAs, respectively, (Figure 4B). The differentially expressed circRNAs were analyzed for expression trend (the expression of circRNA was expressed in Reads of exon model per million mapped reads(RPM), and the data were normalized by the method of log2) to reveal the potential effect mode of circRNA on embryonic muscle development. The differential circRNAs were enriched in 20 trends, of which six were significant (*p* < 0.05) (Figure 4C). The circRNA expression trend timeline is shown in Figure 4D. The significant trends included two upward trends and three downward trends. In total, 225 circRNAs were enriched in the upward trend and 196 circRNAs were enriched in the downward trend (Supplementary Data Sheet 2).

### Weighted gene co-expression network analysis

**Figure 5:**
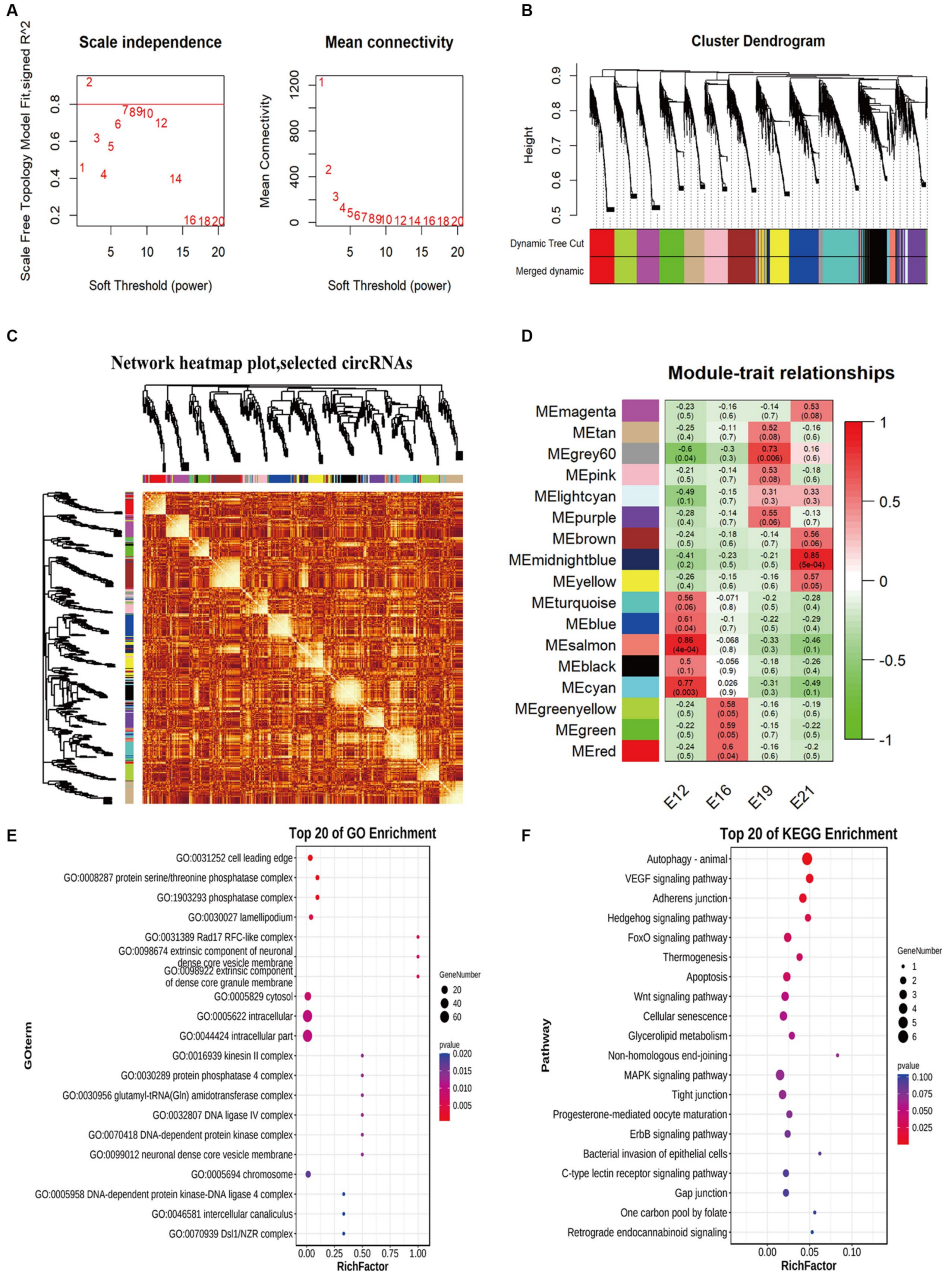
Weighted gene co-expression network analysis of circRNAs. **(A)** The power value curve. **(B)** Module eigenvalue clustering. **(C)** Module gene correlation analysis. **(D)** Correlation analysis of traits. **(E)** Top 20 enriched entries in GO pathway analysis. **(F)** Top 20 enriched entries in KEGG pathway analysis.

The circRNA expression network was constructed to identify potential factors related to muscle development. Therefore, two soft thresholds were chosen to ensure that the weighted gene co-expression network analysis (WGCNA) module conformed to a scale-free distribution (Figure 5A). Thus, we marked the 17 WGCNA modules (ME) identified in this study with different colors (Figure 5B). There were correlations between genes within modules, as well as connections between different modules (Figure 5C). The modules corresponding to different embryonic muscle development stages were significantly different, indicating that circRNAs play different functions at different stages. ME Salmon had the most significant correlations with muscle development (*r* = 0.86, *p* < 0.05) in E12, ME Red in E16 (*r* = 0.60, *p* < 0.05), ME Grey60 in E19 (*r* = 0.73, *p* < 0.05), and ME MidnightBlue in E21 (*r* = 0.85, *p* < 0.05) (Figure 5D). Among the circRNAs of the most significantly associated WGCNA modules, the ten most enriched items in E12 ME Salmon were the cell leading edge, the protein serine/threonine phosphatase complex, the phosphatase complex, the lamellipodium, the *Rad17* RFC-like complex, the extrinsic component of the neuronal dense core vesicle membrane, the extrinsic components of the dense core granule membrane, cytosol, intracellular, and intracellular parts (Figure 5E). A KEGG pathway analysis also revealed that pathways related to cell physiology and organism development were significantly enriched, including the *MAPK* signaling pathway, autophagy, the *VEGF* signaling pathway, the hedgehog signaling pathway, and the *FoxO* signaling pathway (Figure 5F).

### Construction of competitive endogenous RNA networks

**Figure 6:**
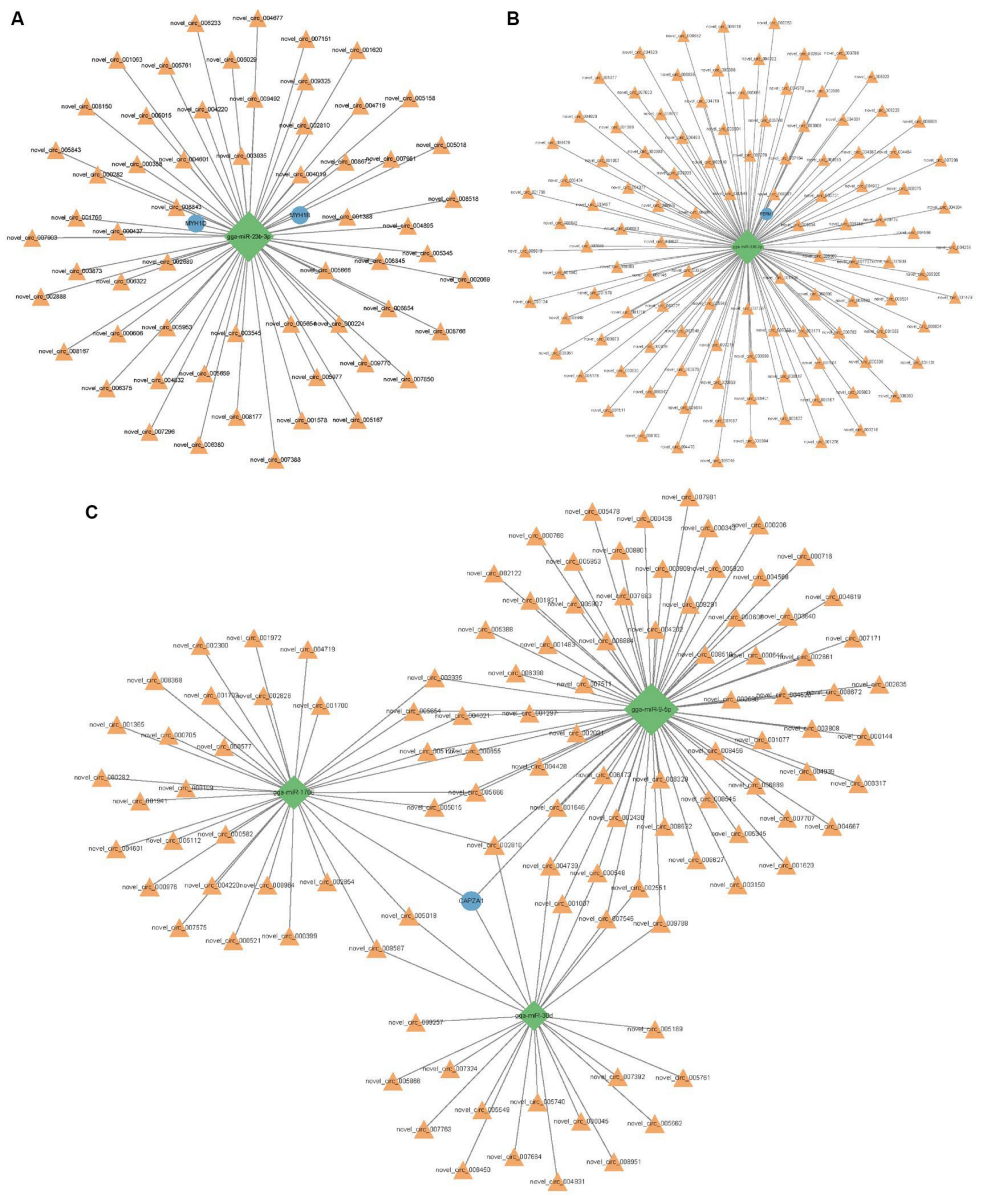
Predicted interactions between circRNA, miRNA and mRNA. **(A)** Networks associated with *MYH1B* and *MYH1D*. **(B)** Networks associated with *PERM1*. **(C)** Networks associated with *CAPZA1*. Orange triangle represents circRNA and blue circle represents gene. The green diamond shape represents the miRNA.

Using Miranda and miRDB, we predicted the associations between circRNAs and microRNAs (miRNAs), as well as between miRNAs and mRNAs. After filtering out irrelevant circRNAs, a competitive endogenous RNA (ceRNA) network was constructed using 754 circRNAs, 68 miRNAs, 2,180 mRNAs, and 3,002 interaction nodes (Supplementary Figure S3). To further explore the effects of circRNAs on skeletal muscle development, this study established the interaction between circRNA and four muscle-related genes, namely *MYH1D*, *MYH1B*, *CAPZA1*, and *PERM1* (Figure 6). Novel_circ_002810, novel_circ_005018, novel_circ_005654, novel_circ_005015, and novel_circ_003935 regulate pathways of nine miRNAs, including gga-miR-23b-3p, gga-miR-9-5p, gga-miR-1706, gga-miR-15c-5p, gga-miR-1644, gga-miR-218-5p, gga-miR-1808, gga-miR-1626-5p, and gga-miR-1773-3p.

### Quantitative verification of candidate circRNAs related to muscle development in Chengkou mountain chicken

**Figure 7:**
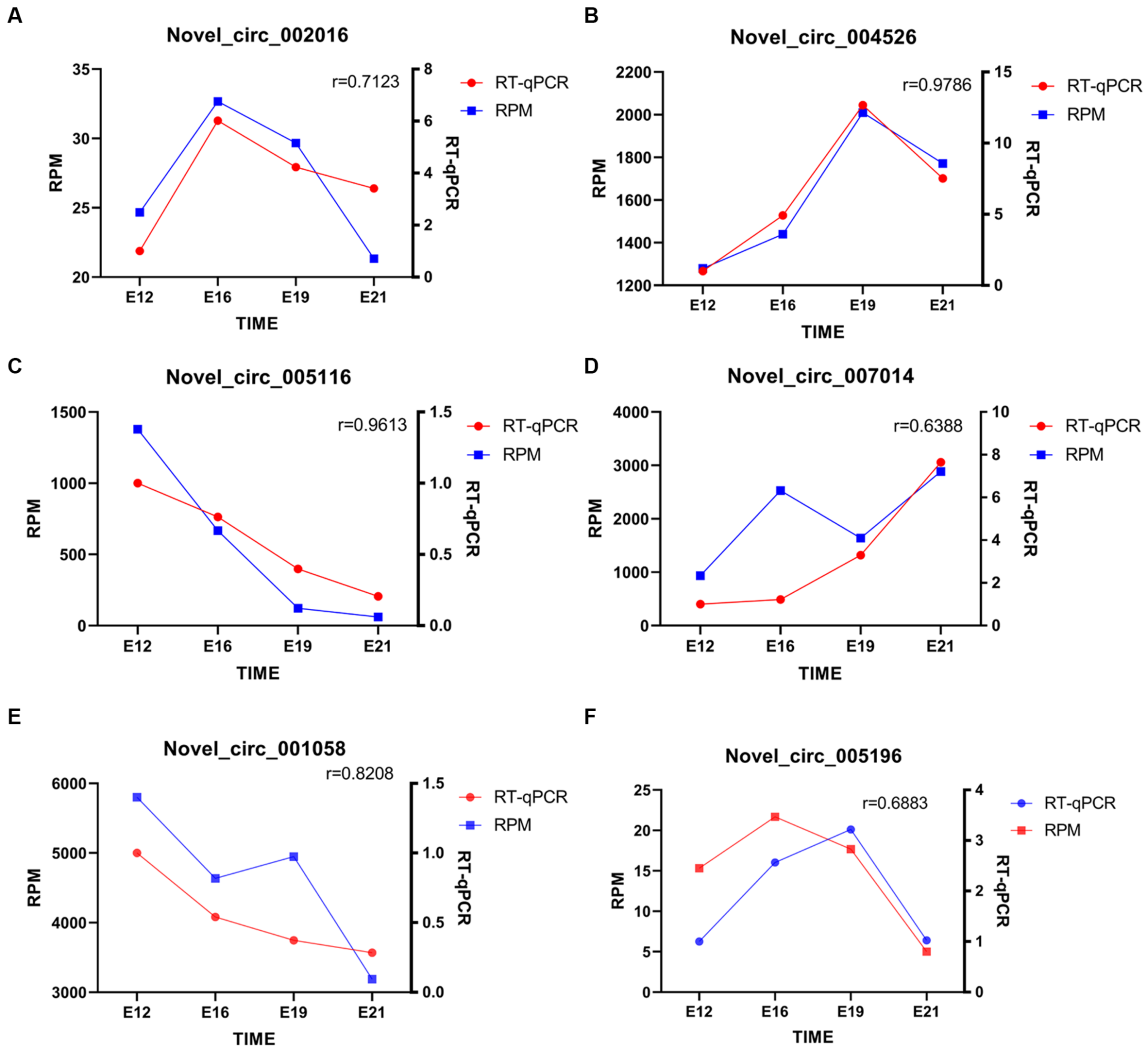
Quantitative analysis of randomly selected circRNAs. **(A–F)** novel_circ_002016, novel_circ_004526, novel_circ_005116, novel_circ_007014, novel_circ_001058, and novel_circ_005196 quantitative analysis. The “*r*” signifies the Pearson correlation coefficient.

Six randomly selected circRNAs were used for RT-qPCR, including novel_circ_002016, novel_circ_004526, novel_circ_005116, novel_circ_007014, novel_circ_001058, and novel_circ_005196. The consistency in expression patterns of circRNAs between circRNA-seq and RT-qPCR results confirms the accuracy of the sequencing (*p* < 0.05) (Figure 7).

## Discussion

Limited research exists on the regulatory mechanisms of growth and development in the local Chengkou mountain chicken breed in China. While our previous study analyzed the role of mRNA in muscle development using transcriptomics, the investigation of circRNAs remains insufficient (19). This study aims to address this gap by focusing on the role and regulation of circRNAs in embryonic muscle development of Chengkou mountain chickens.

The stained muscle sections indicated that the embryonic muscle development pattern of the Chengkou mountain chicken closely resembled that of the Tibetan chicken and other local breeds in China (27, 28). During E12, the muscle fibers were intermixed, and their distinct boundaries were indiscernible. However, by E19, the muscle fibers displayed clear boundaries and were easily identifiable. This insight holds relevance for examining of muscle development in poultry breeds in other regions. Similar to other animal studies, this study identified over 9,000 novel circRNAs, including 917 differentially expressed circRNAs at different stages (29). Our findings are similar to those of Yuan et al. (30), who suggest that chromosome 1 is crucial in skeletal muscle development, as most new circRNAs are derived from it. The comparison of differentially expressed circRNAs between different periods also indicated that different circRNAs regulate myogenesis in different stages. Notably, some of the circRNAs were derived from genes that are highly associated with muscle development, such as *MEF2C*. The *MEF2C* gene affects skeletal muscle growth and development by regulating calcium-mediated carbonic anhydrase III (*CAIII*) expression (31). It is evident that circRNAs are involved in the process of muscle development.

The ME salmon module, the principal WGCNA module linked to muscle development, exhibited the strongest correlation with this trait. Hence, we chose it for analysis of muscle-related pathways and gene enrichment. CircRNAs were found to be enriched in GO and KEGG pathways associated with muscle development, such as significant enrichment in *MAPK* and *Wnt* signaling pathways. The *MAPK* signaling pathway is linked to cell proliferation and differentiation, and both the *Wnt* and *MAPK* signaling pathways are essential for muscle development (32, 33). CircRNAs regulate myogenesis by regulating the mitogen-activated protein *Map3k20* and *JNK/MAPK* signaling pathways (34). Moreover, circRNAs control the growth and maturation of myoblasts by activating the atypical *Wnt5a/Ca*^2+^ pathway and functioning as a miRNA sponge (35). The crucial involvement of circRNAs such as novel_circ_001182 in regulating muscle development via the *MAPK* and *Wnt* signaling pathways underscores the need for further investigation into their regulatory mechanisms in this context.

CircRNAs mainly regulate animal body activities by acting as miRNA sponges (36–38). In this study, we hypothesized that circRNA is important for controlling the growth and development of embryonic muscles. Indeed, the 754 identified circRNAs target 68 known miRNAs (24). These 68 miRNAs and the 2,180 differentially express mRNAs formed the ceRNA network. All transcripts in the ceRNA regulatory network were differentially expressed in the circRNA-miRNA-mRNA pathway, affirming the reliability of the results. Thus, a more comprehensive understanding of the entire regulatory pathway of muscle development can be attained through multi-omics analysis (39). Furthermore, the miRNAs in the network contain regulatory factors related to muscle development. For instance, miR-9-5p stimulates myogenic differentiation through *Dlx3/Myf5*, while miR-15c-5p can regulate muscle generation by activating the *IGF1-PI3K/AKT* signaling pathway (40, 41). The results show that the detected circRNAs may influence muscle growth and development during the embryonic stage by controlling these miRNAs.

The miRDB prediction criteria (scores >80 and miRNA length <2,000) for targeted gene prediction revealed four candidate circRNA-regulated genes related to muscle development, including *MYH1B*, *MYH1D*, *CAPZA1*, and *PERM1* (42). *MYH1B* and *MYH1D* are members of the Myosin Heavy Chain (*MyHC*) gene family, which plays an important role in skeletal muscle growth and development (43, 44). Yu et al. (45) demonstrated the regulatory influence of LncRNA-FKBP1C, a long non-coding RNA, on *MYH1B*, thereby facilitating myoblast differentiation in poultry growth and development. Additionally, during embryonic development, *MYH1D* is significantly enriched in the broiler genome and *MYH1F* is essential for leg muscle growth in Chengkou mountain chicken (19, 46). In this study, the *MyHC* family members were up-regulated over time, indicating that this family is important for muscle fiber growth.

Huang et al. (47) discovered that *CAPZA1* impedes hepatocellular carcinoma (HCC) cell metastasis by managing actin cytoskeleton remodeling in HCC cells, mainly through epithelial-mesenchymal transition. *CAPZA1* encodes the α1 subunit of the actin-conjugated *CapZ*, and its function is related to the assembly of actin filaments (48). In this experiment, the ceRNA network revealed that miR-9-5p suppresses 104 circRNAs, up-regulating *CAPZA1*. This pattern could remodel the actin skeleton, enabling the normal growth and development of muscle (49). The ceRNA analysis demonstrated that decreasing circRNAs up-regulate miR-338-3p and down-regulate *PERM1*. *PERM1* is a muscle-specific regulator that regulates mitochondrial biogenesis and oxidation and enhances the spare respiratory capacity in muscles by enhancing mitochondrial function and vascular formation in skeletal muscles (50). However, whether *CAPZA1* influences skeletal muscle growth through EMT remains unknown. In addition, *PERM1* does not increase the total fiber mass, and the underlying reasons for its regulatory behavior remain unknown. Therefore, the analysis of the ceRNA network clarified that circRNAs are able to influence the activity of genes important for skeletal muscle development by regulating miRNAs, which may have an impact on normal skeletal muscle development.

## Conclusion

This study generated 9,904 circRNAs, including 7,102 “exon” type circRNAs, from 12 cDNA libraries. CircRNA expression is time-specific, and key circRNA-derived genes significantly enriched GO terms and KEGG pathways for cell composition, cell development regulation, and muscle system regulation. This study used sequencing, screening, and circRNA identification in the skeletal muscles of developing Chengkou mountain chicken embryos to identify the macro effects and action pathways of circRNAs on muscle development. Therefore, the results help explore the specific biological functions and mechanisms of key circRNAs in subsequent studies of chicken embryonic muscle development. The numerous differentially expressed circRNAs provide data for subsequent improvement of Chengkou mountain chicken breeding. The discovery of signaling pathways and genes also provides theoretical support for trait improvement of domestic chickens.

## Data availability statement

The datasets presented in this study can be found in online repositories. The names of the repository/repositories and accession number(s) can be found at: https://www.ncbi.nlm.nih.gov/, PRJNA674456.

## Ethics statement

The animal study was approved by Experimental Animal Ethics Review Committee of Southwest University. The ethics review application number is LAC2023-1-0102. The study was conducted in accordance with the local legislation and institutional requirements.

## Author contributions

YZ: Writing – original draft, Writing – review & editing, Validation. HW: Writing – review & editing. XL: Writing – original draft, Validation. CYa: Writing – review & editing. CYu: Writing – review & editing. ZC: Writing – review & editing. AL: Writing – review & editing. QW: Resources, Writing – review & editing. LL: Conceptualization, Funding acquisition, Methodology, Writing – review & editing.

## Footnotes

^1^ http://www.geneontology.org/

^2^ http://sb.cs.cmu.edu/stem/

^3^ cytoscape.org

^4^ mirdb.org

## References

[1] Muaz K Riaz M Akhtar S Park S Ismail A. Antibiotic residues in chicken meat: global prevalence, threats, and decontamination strategies: a review. J Food Prot. (2018) 81:619–27. doi: 10.4315/0362-028X.JFP-17-086, PMID: 29537307

[2] Xu K Zhou H Han C Xu Z Ding J Zhu J. Transcriptomic analysis of *MSTN* knockout in the early differentiation of chicken fetal myoblasts. Genes. (2021) 13:58. doi: 10.3390/genes13010058, PMID: 35052399 PMC8774668

[3] Kim SW Lee JH Park BC Park TS. Myotube differentiation in clustered regularly interspaced short palindromic repeat/Cas9-mediated MyoD knockout quail myoblast cells. Asian Australas J Anim Sci. (2017) 30:1029–36. doi: 10.5713/ajas.16.0749, PMID: 27809462 PMC5495663

[4] Ott MO Bober E Lyons G Arnold H Buckingham M. Early expression of the myogenic regulatory gene, myf-5, in precursor cells of skeletal muscle in the mouse embryo. Development. (1991) 111:1097–107. doi: 10.1242/dev.111.4.1097 1652425

[5] Serralbo O Marcelle C. Migrating cells mediate long-range WNT signaling. Development. (2014) 141:2057–63. doi: 10.1242/dev.107656 24803654

[6] Chal J Pourquié O. Making muscle: skeletal myogenesis in vivo and in vitro. Development. (2017) 144:2104–22. doi: 10.1242/dev.151035, PMID: 28634270

[7] Cinnamon Y Kahane N Bachelet I Kalcheim C. The sub-lip domain—a distinct pathway for myotome precursors that demonstrate rostral-caudal migration. Development. (2001) 128:341–51. doi: 10.1242/dev.128.3.341, PMID: 11152633

[8] Biressi S Molinaro M Cossu G. Cellular heterogeneity during vertebrate skeletal muscle development. Dev Biol. (2007) 308:281–93. doi: 10.1016/j.ydbio.2007.06.006 17612520

[9] Duxson MJ Usson Y Harris AJ. The origin of secondary myotubes in mammalian skeletal muscles: ultrastructural studies. Development. (1989) 107:743–50. doi: 10.1242/dev.107.4.743, PMID: 2483685

[10] Collins CA Olsen I Zammit PS Heslop L Petrie A Partridge TA. Stem cell function, self-renewal, and behavioral heterogeneity of cells from the adult muscle satellite cell niche. Cell. (2005) 122:289–301. doi: 10.1016/j.cell.2005.05.010 16051152

[11] Sanger HL Klotz G Riesner D Gross HJ Kleinschmidt AK. Viroids are single-stranded covalently closed circular RNA molecules existing as highly base-paired rod-like structures. Proc Natl Acad Sci USA. (1976) 73:3852–6. doi: 10.1073/pnas.73.11.3852, PMID: 1069269 PMC431239

[12] Chen R Lei S Jiang T Zeng J Zhou S She Y. Roles of lncRNAs and circRNAs in regulating skeletal muscle development. Acta Physiol. (2020) 228:e13356. doi: 10.1111/apha.13356, PMID: 31365949

[13] Harland R Misher L. Stability of RNA in developing *Xenopus* embryos and identification of a destabilizing sequence in TFIIIA messenger RNA. Development. (1988) 102:837–52. doi: 10.1242/dev.102.4.837, PMID: 2458900

[14] Ouyang H Chen X Wang Z Yu J Jia X Li Z. Circular RNAs are abundant and dynamically expressed during embryonic muscle development in chickens. DNA Res. (2018) 25:71–86. doi: 10.1093/dnares/dsx039, PMID: 29036326 PMC5824844

[15] Legnini I Di Timoteo G Rossi F Morlando M Briganti F Sthandier O. Circ-ZNF609 is a circular RNA that can be translated and functions in myogenesis. Mol Cell. (2017) 66:22–37.e9. doi: 10.1016/j.molcel.2017.02.017, PMID: 28344082 PMC5387670

[16] Wang Y Li M Wang Y Liu J Zhang M Fang X. A Zfp609 circular RNA regulates myoblast differentiation by sponging miR-194-5p. Int J Biol Macromol. (2019) 121:1308–13. doi: 10.1016/j.ijbiomac.2018.09.039, PMID: 30201567

[17] Chen M Liu Q Song M Liu X Huang K Zhong D. CircCLTH promotes skeletal muscle development and regeneration. Epigenetics. (2022) 17:2296–317. doi: 10.1080/15592294.2022.2117115, PMID: 36043316 PMC9665157

[18] Liu J Li M Kong L Cao M Zhang M Wang Y. CircARID1A regulates mouse skeletal muscle regeneration by functioning as a sponge of miR-6368. FASEB J. (2021) 35:e21324. doi: 10.1096/fj.202001992R, PMID: 33421208

[19] Ren L Liu A Wang Q Wang H Dong D Liu L. Transcriptome analysis of embryonic muscle development in Chengkou mountain chicken. BMC Genomics. (2021) 22:431. doi: 10.1186/s12864-021-07740-w, PMID: 34107874 PMC8191012

[20] Kim D Paggi JM Park C Bennett C Salzberg SL. Graph-based genome alignment and genotyping with HISAT2 and HISAT-genotype. Nat Biotechnol. (2019) 37:907–15. doi: 10.1038/s41587-019-0201-4, PMID: 31375807 PMC7605509

[21] Langmead B Salzberg SL. Fast gapped-read alignment with bowtie 2. Nat Methods. (2012) 9:357–9. doi: 10.1038/nmeth.1923, PMID: 22388286 PMC3322381

[22] Memczak S Jens M Elefsinioti A Torti F Krueger J Rybak A. Circular RNAs are a large class of animal RNAs with regulatory potency. Nature. (2013) 495:333–8. doi: 10.1038/nature11928, PMID: 23446348

[23] Zhang B Horvath S. A general framework for weighted gene co-expression network analysis. Stat Appl Genet Mol Biol. (2005) 4:17. doi: 10.2202/1544-6115.1128 16646834

[24] Shi J Li W Liu A Ren L Zhang P Jiang T. MiRNA sequencing of embryonic myogenesis in Chengkou mountain chicken. BMC Genomics. (2022) 23:571. doi: 10.1186/s12864-022-08795-z, PMID: 35948880 PMC9364561

[25] Liu L Ren L Liu A Wang J Wang J Wang Q. Genome-wide identification and characterization of long non-coding RNAs in embryo muscle of chicken. Animals. (2022) 12:1274. doi: 10.3390/ani12101274 35625120 PMC9137640

[26] Liu L Yi J Ray WK Vu LT Helm RF Siegel PB. Fasting differentially alters the hypothalamic proteome of chickens from lines with the propensity to be anorexic or obese. Nutr Diabetes. (2019) 9:13. doi: 10.1038/s41387-019-0081-1, PMID: 30931934 PMC6443654

[27] Pan Z Yang C Zhao R Jiang X Yu C Li Z. Characterization of lncRNA/circRNA-miRNA-mRNA network to reveal potential functional ceRNAs in the skeletal muscle of chicken. Front Physiol. (2022) 13:969854. doi: 10.3389/fphys.2022.969854, PMID: 36246144 PMC9558166

[28] Lei Q Hu X Han H Wang J Liu W Zhou Y. Integrative analysis of circRNA, miRNA, and mRNA profiles to reveal ceRNA regulation in chicken muscle development from the embryonic to post-hatching periods. BMC Genomics. (2022) 23:342. doi: 10.1186/s12864-022-08525-5, PMID: 35505302 PMC9063329

[29] Liu S Wu J Zhang W Jiang H Zhou Y Liu J. Whole-transcriptome RNA sequencing uncovers the global expression changes and RNA regulatory networks in duck embryonic myogenesis. Int J Mol Sci. (2023) 24:16387. doi: 10.3390/ijms242216387 38003577 PMC10671564

[30] Yuan P Zhao Y Li H Li S Fan S Zhai B. CircRNAs related to breast muscle development and their interaction regulatory network in Gushi chicken. Genes. (2022) 13:1974. doi: 10.3390/genes13111974, PMID: 36360215 PMC9689937

[31] Huang H Zhao Y Shang X Ren H Zhao Y Liu X. CAIII expression in skeletal muscle is regulated by Ca^2+^-CaMKII-MEF2C signaling. Exp Cell Res. (2019) 385:111672. doi: 10.1016/j.yexcr.2019.111672, PMID: 31614133

[32] Sun Y Liu WZ Liu T Feng X Yang N Zhou HF. Signaling pathway of MAPK/ERK in cell proliferation, differentiation, migration, senescence and apoptosis. J Recept Signal Transduct Res. (2015) 35:600–4. doi: 10.3109/10799893.2015.1030412, PMID: 26096166

[33] Rim EY Clevers H Nusse R. The Wnt pathway: from signaling mechanisms to synthetic modulators. Annu Rev Biochem. (2022) 91:571–98. doi: 10.1146/annurev-biochem-040320-103615, PMID: 35303793

[34] Yan J Yang Y Fan X Liang G Wang Z Li J. circRNAome profiling reveals circFgfr2 regulates myogenesis and muscle regeneration via a feedback loop. J Cachexia Sarcopenia Muscle. (2022) 13:696–712. doi: 10.1002/jcsm.12859, PMID: 34811940 PMC8818660

[35] Peng S Song C Li H Cao X Ma Y Wang X. Circular RNA SNX29 sponges miR-744 to regulate proliferation and differentiation of myoblasts by activating the Wnt5a/Ca^2+^ signaling pathway. Mol Ther Nucleic Acids. (2019) 16:481–93. doi: 10.1016/j.omtn.2019.03.009, PMID: 31051333 PMC6495097

[36] Zhu M Lian C Chen G Zou P Qin BG. CircRNA FUT10 regulates the regenerative potential of aged skeletal muscle stem cells by targeting HOXA9. Aging. (2021) 13:17428–41. doi: 10.18632/aging.203233, PMID: 34257163 PMC8312443

[37] Yu L Liu Y. circRNA_0016624 could sponge miR-98 to regulate BMP2 expression in postmenopausal osteoporosis. Biochem Biophys Res Commun. (2019) 516:546–50. doi: 10.1016/j.bbrc.2019.06.087 31235259

[38] Ye Y Zhang L Hu T Yin J Xu L Pang Z. CircRNA_103765 acts as a proinflammatory factor via sponging miR-30 family in Crohn’s disease. Sci Rep. (2021) 11:565. doi: 10.1038/s41598-020-80663-w, PMID: 33436852 PMC7804428

[39] Stanberry L Mias GI Haynes W Higdon R Snyder M Kolker E. Integrative analysis of longitudinal metabolomics data from a personal multi-omics profile. Metabolites. (2013) 3:741–60. doi: 10.3390/metabo3030741, PMID: 24958148 PMC3901289

[40] Li Z Cai B Abdalla BA Zhu X Zheng M Han P. LncIRS1 controls muscle atrophy via sponging miR-15 family to activate IGF1-PI3K/AKT pathway. J Cachexia Sarcopenia Muscle. (2019) 10:391–410. doi: 10.1002/jcsm.12374, PMID: 30701698 PMC6463472

[41] Dong L Wang M Gao X Zheng X Zhang Y Sun L. miR-9-5p promotes myogenic differentiation via the Dlx3/Myf5 axis. PeerJ. (2022) 10:e13360. doi: 10.7717/peerj.13360, PMID: 35529491 PMC9074878

[42] Chen Y Wang X. miRDB: an online database for prediction of functional microRNA targets. Nucleic Acids Res. (2020) 48:D127–31. doi: 10.1093/nar/gkz757, PMID: 31504780 PMC6943051

[43] Dou M Yao Y Ma L Wang X Shi X Yang G. The long noncoding RNA MyHC IIA/X-AS contributes to skeletal muscle myogenesis and maintains the fast fiber phenotype. J Biol Chem. (2020) 295:4937–49. doi: 10.1074/jbc.RA119.010498, PMID: 32152230 PMC7152763

[44] Fazarinc G Vrecl M Poklukar K Škrlep M Batorek-Lukač N Brankovič J. Expression of myosin heavy chain and some energy metabolism-related genes in the longissimus dorsi muscle of Krškopolje pigs: effect of the production system. Front Vet Sci. (2020) 7:533936. doi: 10.3389/fvets.2020.533936, PMID: 33062658 PMC7530236

[45] Yu JA Wang Z Yang X Ma M Li Z Nie Q. LncRNA-FKBP1C regulates muscle fiber type switching by affecting the stability of MYH1B. Cell Death Discov. (2021) 7:73. doi: 10.1038/s41420-021-00463-7, PMID: 33837177 PMC8035166

[46] Tan X Liu R Li W Zheng M Zhu D Liu D. Assessment the effect of genomic selection and detection of selective signature in broilers. Poult Sci. (2022) 101:101856. doi: 10.1016/j.psj.2022.101856, PMID: 35413593 PMC9018145

[47] Huang D Cao L Zheng S. CAPZA1 modulates EMT by regulating actin cytoskeleton remodelling in hepatocellular carcinoma. J Exp Clin Cancer Res. (2017) 36:13. doi: 10.1186/s13046-016-0474-0, PMID: 28093067 PMC5240199

[48] Isenberg G Aebi U Pollard TD. An actin-binding protein from *Acanthamoeba* regulates actin filament polymerization and interactions. Nature. (1980) 288:455–9. doi: 10.1038/288455a0, PMID: 6893736

[49] Lu Y Huang D Wang B Zheng B Liu J Song J. FAM21C promotes hepatocellular carcinoma invasion and metastasis by driving actin cytoskeleton remodeling via inhibiting capping ability of CAPZA1. Front Oncol. (2021) 11:809195. doi: 10.3389/fonc.2021.809195 35096613 PMC8793146

[50] Cho Y Hazen BC Gandra PG Ward SR Schenk S Russell AP. Perm1 enhances mitochondrial biogenesis, oxidative capacity, and fatigue resistance in adult skeletal muscle. FASEB J. (2016) 30:674–87. doi: 10.1096/fj.15-276360, PMID: 26481306 PMC4714556

