# Supplementary figures and images for: Genome-wide characteristics and potential functions of circular RNAs from the embryo muscle development in Chengkou mountain chicken
Source: Front Vet Sci. 2024 May 30;11:1375042. doi: 10.3389/fvets.2024.1375042 (PMC11171140; doi:10.3389/fvets.2024.1375042)

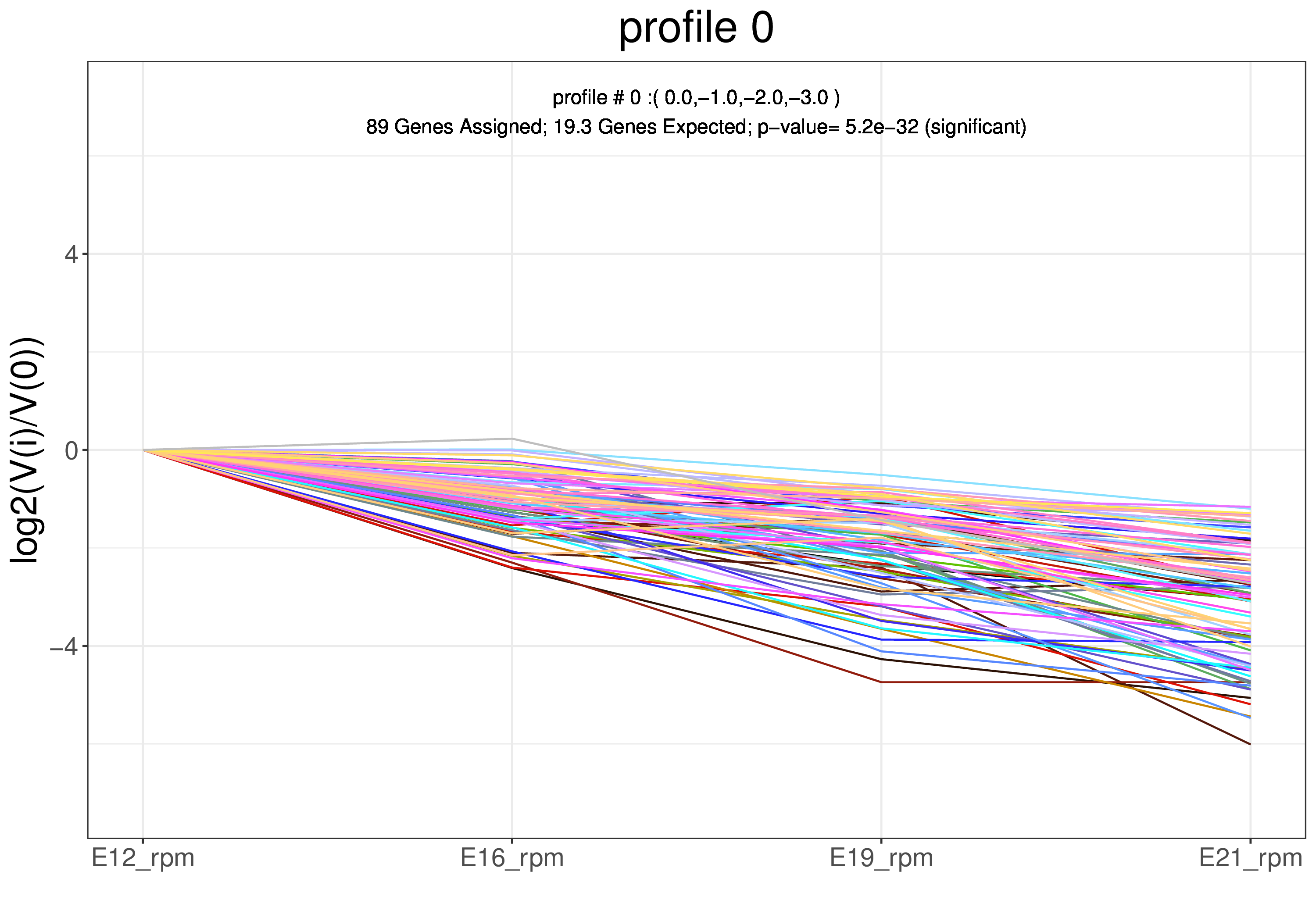

Supplement: Supplementary file 1 [file Data_Sheet_1.ZIP › Supplement 3/down/profile0.png]

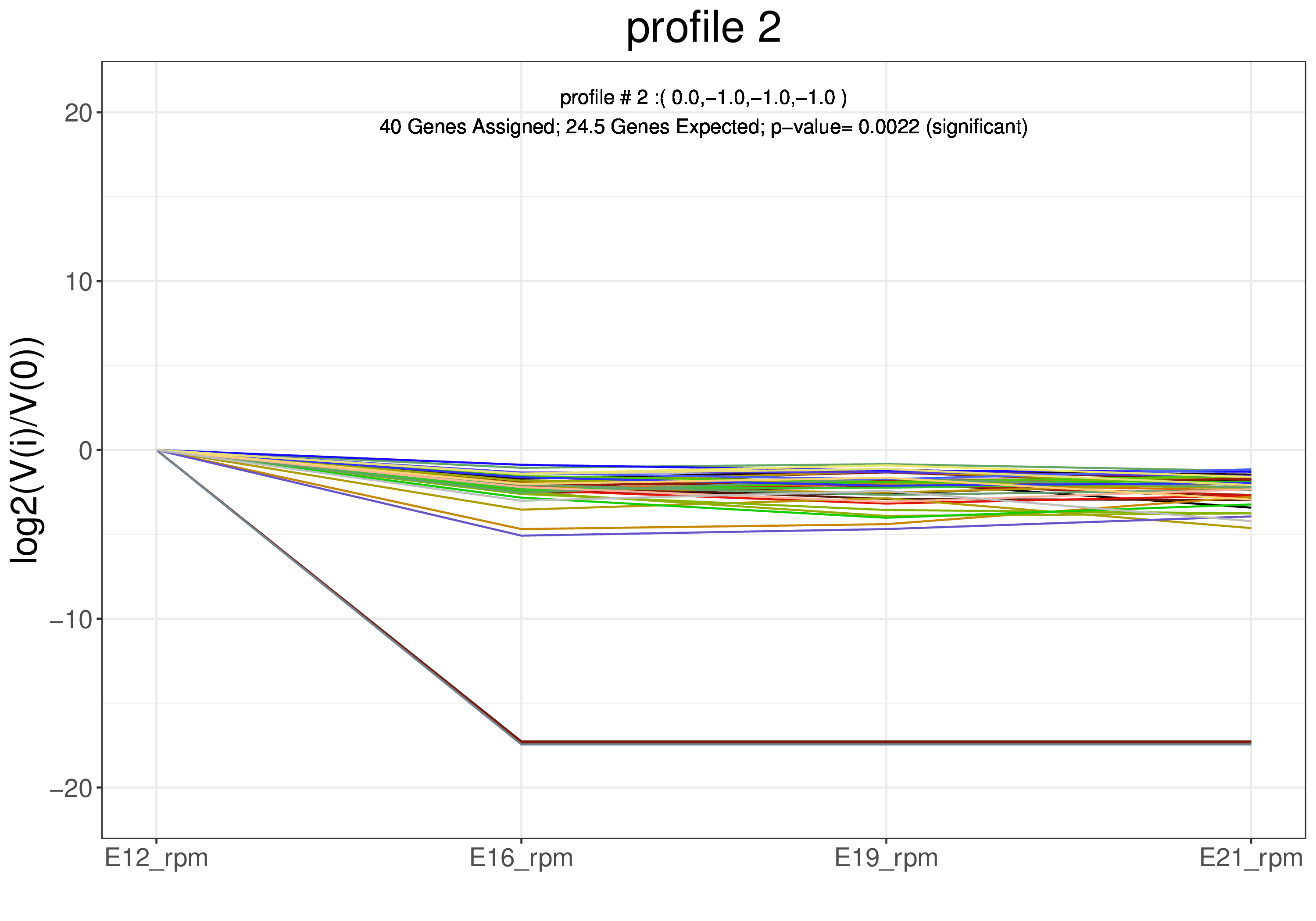

Supplement: Supplementary file 1 [file Data_Sheet_1.ZIP › Supplement 3/down/profile2.png]

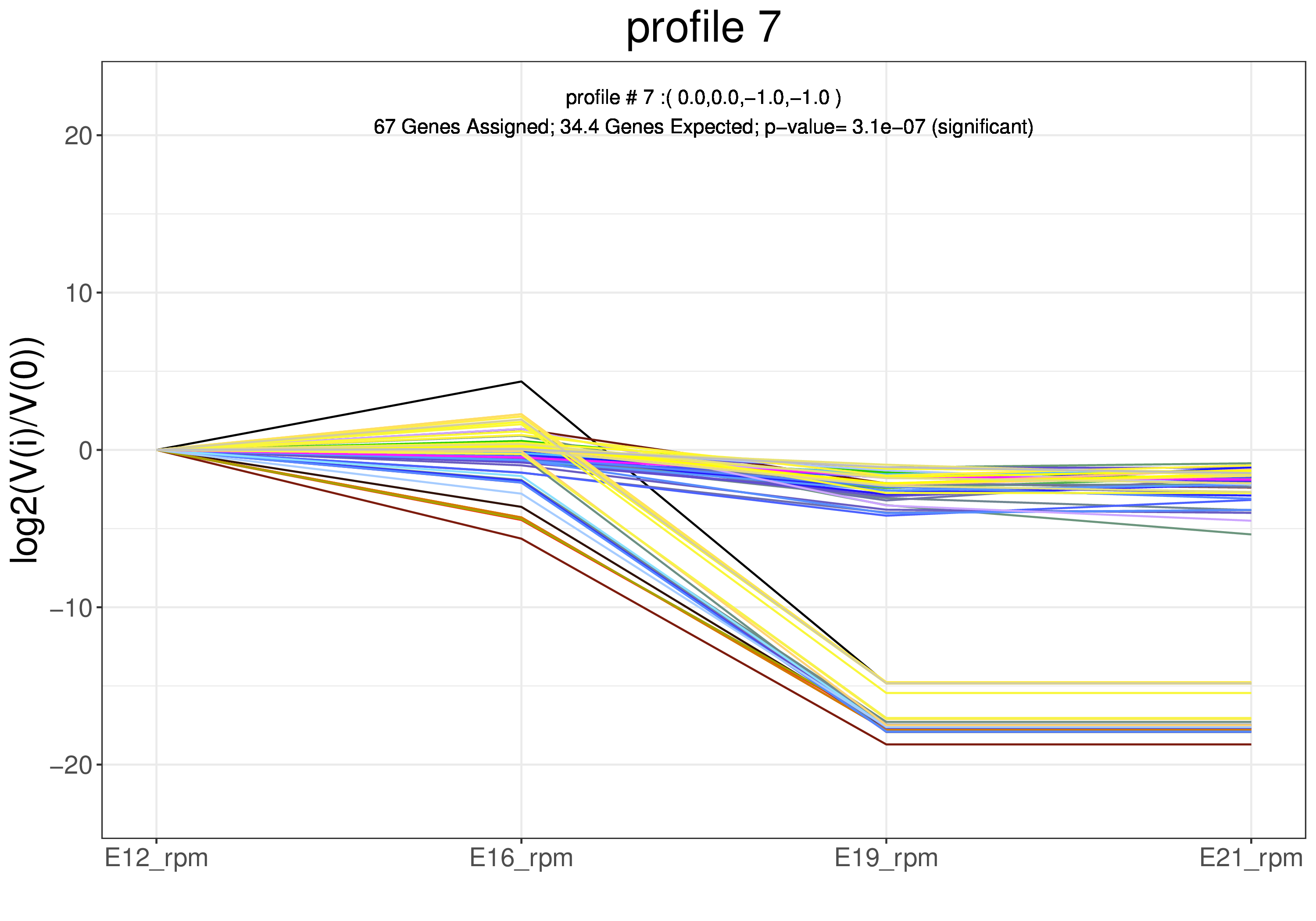

Supplement: Supplementary file 1 [file Data_Sheet_1.ZIP › Supplement 3/down/profile7.png]

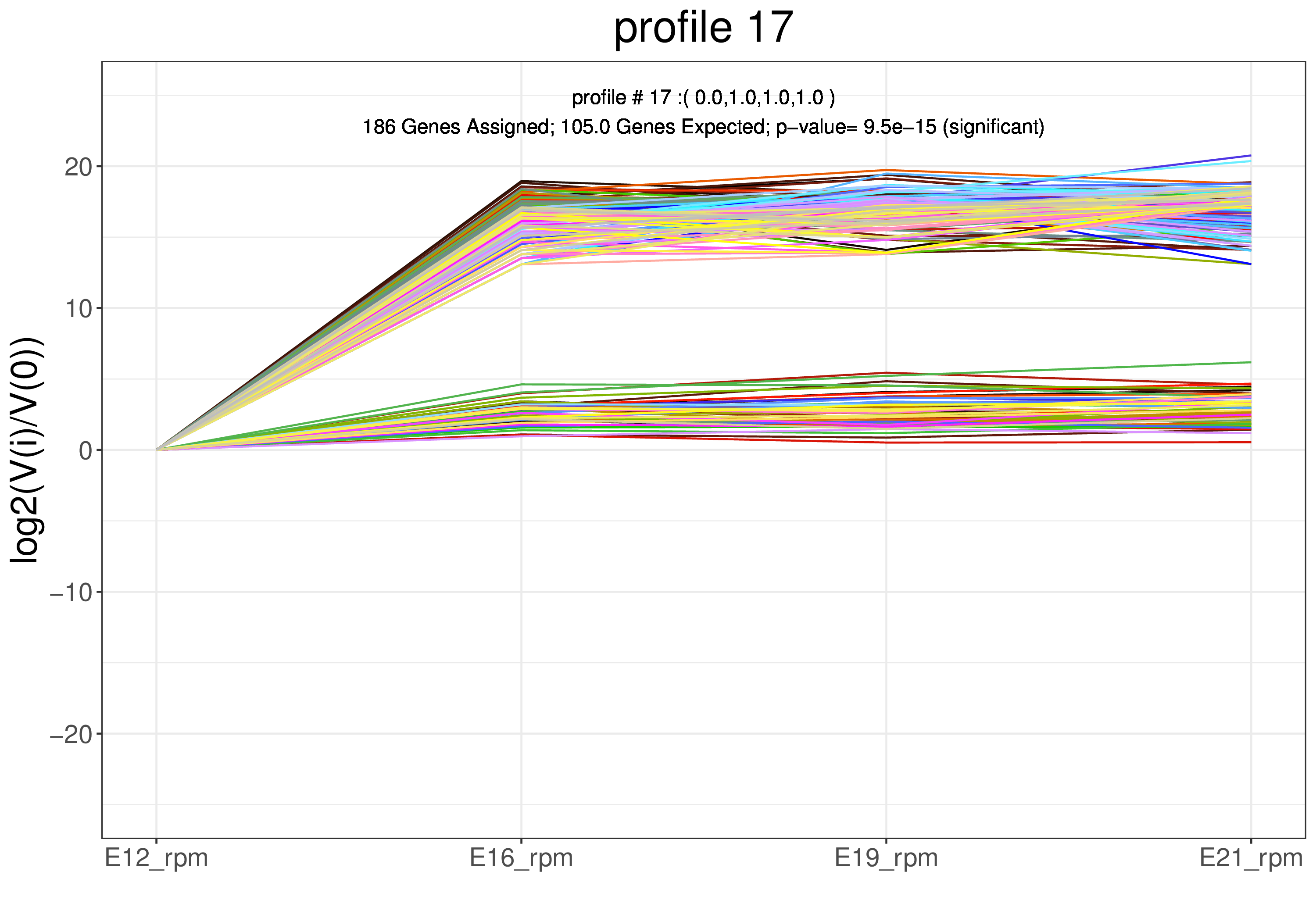

Supplement: Supplementary file 1 [file Data_Sheet_1.ZIP › Supplement 3/up/profile17.png]

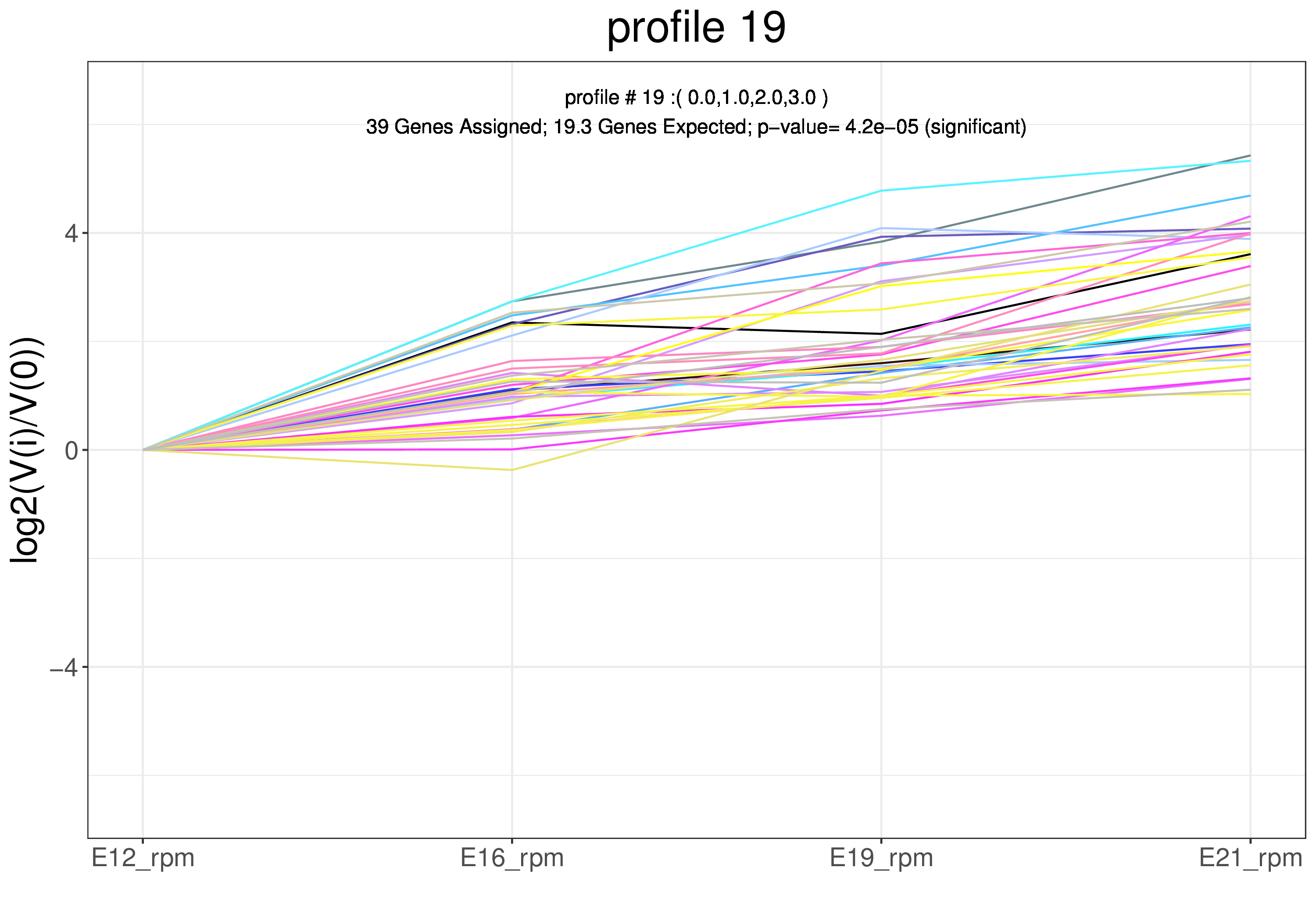

Supplement: Supplementary file 1 [file Data_Sheet_1.ZIP › Supplement 3/up/profile19.png]
